# A randomized placebo-controlled trial of delayed-release dimethyl fumarate in patients with relapsing-remitting multiple sclerosis from East Asia and other countries

**DOI:** 10.1186/s12883-018-1220-3

**Published:** 2019-01-07

**Authors:** Takahiko Saida, Takashi Yamamura, Takayuki Kondo, Jang Yun, Minhua Yang, Jie Li, Lalitha Mahadavan, Bing Zhu, Sarah I. Sheikh

**Affiliations:** 1 Kansai Multiple Sclerosis Centre, Kyoto Min-iren Central Hospital, Nishinokyo-Kasuga-cho 16-44-409, Nakakyo-ku, Kyoto, 604-8453 Japan; 2 NCNP, National Center Hospital, Tokyo, Japan; 3 Kansai Medical University Medical Center, Osaka, Japan; 4 Biogen, Cambridge, MA USA; 5 Sanofi, Cambridge, MA USA; 6 Faculty of Pharmaceutical Medicine, London, UK

**Keywords:** Delayed-release dimethyl fumarate, East Asia, Japan, Magnetic resonance imaging, Multiple sclerosis, Randomized clinical trial

## Abstract

**Background:**

Delayed-release dimethyl fumarate (DMF) has demonstrated efficacy and a favorable benefit-risk profile in phase 2 and 3 studies that enrolled predominantly white patients with relapsing-remitting multiple sclerosis (RRMS). In this study (APEX, Part I), we evaluated the efficacy/safety outcomes of DMF in a predominantly East Asian population of patients with RRMS.

**Methods:**

In this 24-week, randomized, double-blind, placebo-controlled phase 3 study, 225 patients, 142 of which were East Asian (63.4%), were enrolled: Japan (*n* = 114), South Korea (*n* = 20), Taiwan (*n* = 8), the Czech Republic (*n* = 42), and Poland (*n* = 40). Key exclusion criteria included diagnosis of neuromyelitis optica spectrum disorder. Stratified by country, patients were randomized 1:1 to receive DMF 240 mg twice daily or placebo. Clinical assessments, including neurological examination and EDSS scoring, were conducted at baseline and at weeks 12 and 24.

**Results:**

A total of 213 patients (95.1%) completed the study. From weeks 12 – 24, the total number of new gadolinium-enhancing (Gd^+^) lesions was reduced by 84% (*p* < 0.0001) in DMF compared with placebo. For the secondary endpoint, from baseline to week 24, the total number of new Gd^+^ lesions was reduced by 75% and the mean number of new/newly enlarging T2 hyperintense lesions was reduced by 63% (both *p* < 0.0001). Flushing and flushing-related symptoms, and gastrointestinal events were adverse events related to DMF treatment. Efficacy and safety results in the Japanese subgroup and the East Asian subgroup (which included patients from Japan, Taiwan, and South Korea) were consistent with the overall study population.

**Conclusion:**

The strong efficacy and favorable benefit-risk profile of DMF extends to Japanese, and more broadly, East Asian patients with RRMS.

**Trial registration:**

This trial is registered on ClinicalTrials.gov (identifier: NCT01838668), April 20, 2013 (retrospectively registered). The registration can be found at the following URL: https://clinicaltrials.gov/ct2/show/NCT01838668

**Electronic supplementary material:**

The online version of this article (10.1186/s12883-018-1220-3) contains supplementary material, which is available to authorized users.

## Background

Delayed-release dimethyl fumarate (DMF) 240 mg twice daily (BID) is an oral therapy recently approved in the United States, Europe, and other regions for the treatment of relapsing multiple sclerosis (MS). In pivotal clinical studies (DEFINE/CONFIRM), DMF demonstrated significant efficacy on clinical and neuroradiological measures of disease activity and a favorable benefit-risk profile in patients with relapsing-remitting MS (RRMS) [1, 2]. The study populations were predominantly white (79% in DEFINE and 84% in CONFIRM) and contained only 10% or fewer patients of Asian ethnicity. A pharmacokinetic study evaluated 2 different dosing regimens of DMF (120 mg BID and 240 mg BID) in Chinese, Japanese, and white adult healthy volunteers, and indicated that the pharmacokinetic behavior of DMF was similar across the 3 ethnic groups and was consistent with the exposure observed in other studies [3]. However, there is still relatively little information available on DMF efficacy and safety in Asian patients with MS, especially those from the East-Asia region.

In Japan, compared to Western countries, the prevalence of MS and the proportion of patients with primary and secondary progressive clinical courses are significantly lower, and cerebellar symptoms are relatively infrequent [4, 5]. However, MS prevalence has increased significantly in recent years in East Asia, particularly in Japan, where the current estimated prevalence is 10/100,000 [6–8]. This increase may be related to increased awareness of the disease, improved diagnostic criteria, and/or lifestyle changes. Although the pathophysiology of MS is considered similar in white and Asian populations, differences in genetic and environmental factors may have an impact on the efficacy and safety of MS therapeutics [7, 9]. Neuromyelitis optica spectrum disorder (NMOSD) is an autoimmune disease that targets the optic nerves and spinal cord. Although it has a similar clinical presentation, it is distinct from MS in terms of its pathological causes and response to disease-modifying treatments [10]. The ratio of NMOSD to MS is much higher in Asian countries compared with Western countries [9], thus it is important to use criteria to exclude patients with NMOSD from clinical trials of MS in predominantly Asian populations.

In the present study, APEX Part 1, we evaluated the efficacy/safety profile of DMF 240 mg BID compared to placebo over 6 months of treatment in patients with RRMS from East Asia and other countries which specifically excluded patients with NMOSD (ClinicalTrials.gov identifier: NCT01838668).

## Methods

### Patients

Primary recruitment was for patients aged 18–55 years with ethnic origins from East Asia (Japan, South Korea, or Taiwan). To maintain the power of the study and to allow for subgroup comparison, enrollment was expanded to patients from Eastern Europe (the Czech Republic and Poland). Key inclusion criteria included an RRMS diagnosis per McDonald criteria [11], Expanded Disability Status Scale (EDSS) [12] score of 0–5.0, brain magnetic resonance imaging (MRI) results consistent with MS, and disease activity as evidenced by ≥1 relapse within the 12 months before randomization or the presence of gadolinium-enhancing (Gd^+^) lesions on brain MRI scans within 6 weeks before randomization. Key exclusion criteria were progressive forms of MS, diagnosis or history of NMOSD or a history of positive tests for anti–aquaporin 4 antibodies [10, 13], relapse within 50 days before randomization or lack of stabilization from a prior relapse, and exposure to contraindicated medications within specific time periods (see Additional file 1 Additional methods for details).

### Study design

APEX is a randomized phase 3 study conducted in 2 parts: a 24-week, double-blind, placebo-controlled portion (Part I, which is complete), followed by an open-label extension (Part II, which is ongoing). In Part I, patients were randomly assigned in a 1:1 ratio to receive oral DMF 240 mg BID or matching placebo for 24 weeks; DMF was administered at a reduced dose (120 mg BID) during the first week of the study to enhance tolerability. Randomization was performed using a centralized interactive voice/web response system (Endpoint Clinical Inc., San Francisco, CA) and was stratified by country (for more details, see Additional file 1 Additional methods).

Patients, their families, and all study staff were blinded to patient treatment assignments. In addition, separate study personnel were designated to treat patients and to conduct efficacy and relapse assessments. Patients were instructed not to take a dose of study treatment within 4 h before their scheduled appointment to prevent any drug-induced reactions necessitating unblinding of study personnel.

Clinic visits, brain MRI scans (with and without Gd), and laboratory/safety evaluations were conducted every 4 weeks (±5 days) during the treatment period. In patients who were treated with intravenous pulse methylprednisolone (IVMP) for relapse, MRI was performed just prior to initiation of IVMP. Any MRI scheduled to occur within the 28 days following IVMP treatment was omitted. Participating sites were equipped with 1.5T or 3.0T MRI scanners. For each patient, scans were performed on the same scanner and head coil throughout the study. The scanning protocol consisted of 8 different MRI acquisitions, 3 scouts and 5 sequences. The fast localizer scan (3 PLANE), true mid-line sagittal scan, fast axial scan for repositioning, Proton Density-weighted (PDW) sequence, T2-weighted (T2W) sequence, and T1-weighted (T1W) pre-Gd sequence, were performed prior to Gd injection. After Gd injection, Turbo FLAIR sequence was performed immediately within the 10-min waiting period, and T1-weighted post-Gd sequence was performed after the 10-min waiting period. To ensure the quality and consistency of MRI measurements, the MRI capability of all investigational sites was validated and sequence parameters were provided by a central MRI reading center (NeuroRx Research). MRI technicians at study sites and at the central MRI reading center were blinded to patients’ treatment assignments. Clinical assessments, including neurological examinations and EDSS scoring, were completed at baseline and at weeks 12 and 24.

Relapses were defined as new or recurrent neurologic symptoms not associated with fever or infection, lasting ≥24 h, and accompanied by new neurological findings, assessed objectively. The treatment for relapse was 3 to 5 days of intravenous methylprednisolone, dosed at 1000 mg/day. Systemic steroid treatment was disallowed except for relapse treatment.

### Endpoints

The primary endpoint for the study was the total number of new Gd^+^ lesions on brain MRI scans from weeks 12–24, calculated as the sum of new Gd^+^ lesions found from scans performed at weeks 12, 16, 20, and 24. Secondary endpoints included: (1) the total number of new Gd^+^ lesions from baseline to week 24, calculated as the sum of the new Gd^+^ lesions from 6 brain MRI scans performed at weeks 4, 8, 12, 16, 20, and 24, and (2) the number of new/newly enlarging T2 hyperintense lesions at week 24 compared with baseline (these lesions were identified by comparing MRI scans at week 24 to scans at baseline). Tertiary endpoints included standard safety measurements, annualized relapse rate (ARR) over 24 weeks, and the proportion of patients with relapse over 24 weeks.

### Statistical analysis

Efficacy and safety analyses were based on the intention-to-treat (ITT) population, defined as all patients who were randomized and received ≥1 dose of study medication. Data obtained after patients switched to an alternative MS medication were excluded. Subgroup analyses were performed for patients from Japan only (Japanese subgroup) and patients from Japan, South Korea, and Taiwan (East Asian subgroup). The Eastern European subgroup is provided for reference.

Assuming the mean (standard deviation) for the total number of new Gd^+^ lesions from 4 MRI scans in weeks 12–24 for the placebo group is 4.5 (7.8) and for the DMF group is 1.8 (3.8), a sample size of 101 patients per cohort was determined to have ~ 80% power to detect a treatment effect of a ≥ 60% reduction in primary endpoint, comparing the mean of the placebo group with the mean of the DMF group. The mean and standard deviation estimate for the placebo group were determined based on data from the phase 2b study of DMF [14]. A 15% dropout rate also was assumed and accounted for in the calculation.

The primary and secondary endpoints were analyzed using negative binomial regression, adjusted for baseline values and region (East Asian vs. Other). Three sensitivity analyses were conducted for the primary endpoint, as described in Additional file 1 Additional methods.

ARR over 24 weeks was analyzed using negative binomial regression. The proportion of patients with relapse over 24 weeks was estimated from the Kaplan-Meier curve of time to first relapse and analyzed using a Cox proportional hazards model. Both relapse endpoints were adjusted for baseline EDSS score (≤2.0, > 2.0), baseline age (< 40 years, ≥40 years), region (East Asia vs Other), and number of relapses in the year before study entry.

Safety analyses were summarized using descriptive statistics. Adverse events (AEs) were evaluated based on treatment emergence. AEs of special interest are defined in Additional file 1 Additional methods.

## Results

### Patients

**Fig. 1:**
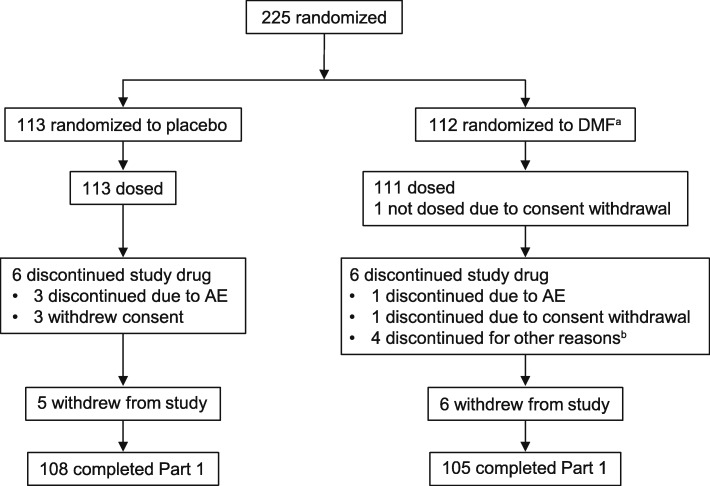
Patient disposition. *AE* adverse event. ^a^DMF, delayed-release DMF. ^b^Reasons for discontinuation documented by investigators were gastrointestinal intolerance for study medication, moved, plasmapheresis, and positive urine pregnancy test

A total of 224 patients at 54 sites were randomized and received ≥1 dose of study treatment, including 113 in the placebo group and 111 in the DMF group; 2 patients (both in the placebo group) switched to an alternative MS medication during the study (Fig. 1). Approximately half of the total patients (51%) were from Japan and approximately two-thirds (63%) were from East Asia (Japan, South Korea, or Taiwan). The Eastern European subgroup comprised the remainder (37%) of the population. A total of 213 patients (95% of the ITT population) completed the study. Baseline demographic/disease characteristics were generally well balanced between the treatment arms, as well as in the Japanese and East Asian subgroups (Table 1). A total of 57% of patients had previously been treated with any MS therapy. IFN β-1a was the most commonly used (25%), followed by IFN β-1b (22%), fingolimod (7%), GA (5%), and natalizumab (2%).

**Table 1:** Baseline demographic and disease characteristics of the ITT population, East Asian subgroup, Japanese subgroup, and Eastern European Subgroup

	ITT		East Asian subgroup		Japanese subgroup		Eastern European subgroup	
Characteristic	Placebo *n* = 113	DMF^a^ *n* = 111	Placebo *n* = 72	DMF^a^ *n* = 70	Placebo *n* = 58	DMF^a^ *n* = 56	Placebo *n* = 41	DMF^a^ *n* = 41
Age (years), mean (SD)	36.0 (7.5)	37.3 (8.3)	36.6 (7.9)	37.5 (8.4)	36.4 (7.2)	38.4 (8.2)	35.0 (6.6)	37.0 (8.2)
Female (%)	74	70	79	79	79	79	66	56
BMI (kg/m^2^), mean (SD)	23.0 (4.3)	23.2 (4.1)	21.7 (3.4)	22.1 (3.3)	21.5 (3.6)	22.1 (3.5)	25.4 (4.7)	25.1 (4.6)
Country, *n* (%)								
Japan	58 (51)	56 (50)	58 (81)	56 (80)	58 (100)	56 (100)	0	0
South Korea	10 (9)	10 (9)	10 (14)	10 (14)	0	0	0	0
Taiwan	4 (4)	4 (4)	4 (6)	4 (6)	0	0	0	0
Czech Republic	21 (19)	21 (19)	0	0	0	0	21 (51)	21 (51)
Poland	20 (18)	20 (18)	0	0	0	0	20 (49)	20 (49)
Region								
East Asia	72 (64)	70 (63)	72 (100)	70 (100)	58 (100)	56 (100)	0	0
Eastern Europe	41 (36)	41 (37)	0	0	0	0	41 (100)	41 (100)
Relapses in prior year, mean (SD)	1.4 (0.7)	1.4 (0.7)	1.3 (0.6)	1.4 (0.7)	1.3 (0.6)	1.5 (0.7)	1.5 (0.8)	1.5 (0.6)
Relapses in last 3 years, mean (SD)	2.3 (1.5)	2.5 (1.7)	2.3 (1.6)	2.6 (1.8)	2.3 (1.7)	2.7 (1.9)	2.3 (1.3)	2.3 (1.4)
Time since last relapse (months), mean (SD)	6.4 (6.5)	5.5 (4.7)	7.0 (7.7)	5.9 (5.6)	7.5 (8.4)	6.0 (6.0)	5.2 (3.6)	4.7 (2.4)
EDSS score, mean (SD)	1.9 (1.3)	2.2 (1.3)	1.8 (1.3)	1.9 (1.3)	1.8 (1.3)	1.9 (1.3)	2.1 (1.2)	2.6 (1.2)
EDSS score, median (min, max)	2.0 (0, 5)	2.0 (0, 5)	1.8 (0, 5)	2.0 (0, 4.5)	1.5 (0, 5)	2.0 (0, 4.5)	2.0 (0, 5)	2.5 (1, 5)
Any prior treatment for MS, *n* (%)	64 (57)	63 (57)	35 (49)	40 (57)	31 (53)	31 (55)	29 (71)	23 (56)
Number of Gd^+^ lesions, mean (SD)	1.5 (3.2)	1.6 (4.9)	1.8 (3.6)	1.3 (2.7)	1.6 (3.3)	1.3 (2.6)	1.1 (2.1)	2.0 (7.3)
T2 hyperintense lesion volume (cm^3^), mean (SD)	8.2 (10.3)	6.7 (7.7)	8.6 (9.5)	6.2 (7.5)	8.1 (8.9)	5.7 (7.3)	7.3 (11.5)	7.6 (8.1)

*Abbreviations: ITT* intention-to-treat, *SD* standard deviation, *BMI* body mass index, *EDSS* Expanded Disability Status Scale, *MS* multiple sclerosis, *Gd*^*+*^ gadolinium-enhancing

^a^DMF, delayed-release DMF

### Efficacy

DMF reduced the mean number of new Gd^+^ lesions in weeks 12–24 (primary endpoint) by 84% in the overall ITT population (0.5 vs 3.3), 85% in the Japanese subgroup, and 81% in the East Asian subgroup, compared with placebo (all *p* < 0.0001; Table 2). In the Eastern European subgroup, there was an 87% percentage reduction of number of new Gd^+^ lesions in weeks 12–24. Results of the 3 sensitivity analyses were consistent with the primary analysis, as described in Additional file 1 Additional methods. Analysis of patients in the efficacy-evaluable population (defined as patients who had no missing MRI scans at weeks 12, 16, 20, and 24) showed a similar reduction of 88% (95% CI, 79.2–93.2%).

**Table 2:** MRI and clinical endpoints in the ITT population, East Asian subgroup, Japanese subgroup, and Eastern European subgroup

	ITT		East Asian subgroup		Japanese subgroup		Eastern European subgroup	
Endpoint, *n* (%)	Placebo *n* = 113	DMF^a^ *n* = 111	Placebo *n* = 72	DMF^a^ *n* = 70	Placebo *n* = 58	DMF^a^ *n* = 56	Placebo *n* = 41	DMF^a^ *n* = 41
Adjusted mean total number of new Gd^+^ lesions from weeks 12–24								
Mean	3.3	0.5	3.2	0.6	2.7	0.4	5.1	1.8
Percentage reduction vs placebo		84		82		85		87
(95% CI)		(73.4–89.9)		(64.7–90.3)		(69.5–92.9)		(71.5–93.7)
*p* value		< 0.0001		< 0.0001		< 0.0001		NT
Adjusted mean total number of new Gd^+^ lesions from baseline to week 24								
Mean	4.8	1.2	4.9	1.2	4.3	0.9	7.0	4.8
Percentage reduction vs placebo		75		76		78		73
(95% CI)		(63.4–83.3)		(60.5–85.6)		(63.1–87.4)		(48.5–85.3)
*p* value		< 0.0001		< 0.0001		< 0.0001		NT
Adjusted mean number of new/newly enlarging T2 hyperintense lesions at week 24 compared vs baseline								
Mean	4.3	1.6	3.9	1.6	3.7	1.4	4.9	1.5
Percentage reduction vs placebo		63		58		63		70
(95% CI)		(47.5–74.2)		(35.4–73.1)		(40.2–77.4)		(44.1–83.7)
*p* value		< 0.0001		< 0.0001		< 0.0001		NT
ARR at week 24								
Adjusted ARR	0.65	0.45	1.01	0.53	1.17	0.60	0.20	0.39
Percentage reduction vs placebo		31		47		48		−100.2
(95% CI)		(−10.8, 56.8)		(8.3–69.7)		(7.4–71.2)		(− 525.6–36.0)
*p* value		0.1251		NT		NT		NT
Proportion of patients relapsed at week 24								
Estimated proportion relapsed	0.30	0.21	0.41	0.24	0.45	0.26	0.12	0.17
Percentage reduction vs placebo		42		53		56		−38.6
(95% CI)		(0.7–66.5)		(11.7–75.0)		(13.1–77.4)		(−364.0–58.6)
*p* value		0.0472		NT		NT		NT

*Abbreviations: MRI* magnetic resonance imaging, *ITT* intention-to-treat, *Gd*^*+*^ gadolinium-enhancing, *CI* confidence interval, *ARR* annualized relapse rate, *NT*, not tested

^a^DMF, delayed-release DMF

On secondary endpoints, DMF reduced the total number of new Gd^+^ lesions from baseline to week 24 by 75% in the ITT population (1.2 vs 4.8), 78% in the Japanese subgroup, and 76% in the East Asian subgroup, compared with placebo (all *p* < 0.0001). In the Eastern European subgroup, there was a 73% percentage reduction of number of new Gd^+^ lesions from baseline to week 24. In addition, DMF reduced the number of new/newly enlarging T2 hyperintense lesions at week 24 by 63% in the ITT population (1.6 vs 4.3), 63% in the Japanese subgroup, and 58% in the East Asian subgroup, compared with placebo (all *p* < 0.0001; Table 2).

Consistent with the above findings, the proportions of DMF-treated patients that had no new Gd^+^ lesions or no new/newly enlarging T2 lesions over the study period were markedly increased, and the proportion of DMF-treated patients with ≥3 lesions over the study period was markedly decreased, compared to the placebo group (Table 3).

**Table 3:** Number of lesions on MRI in the ITT population, East Asian subgroup, Japanese subgroup, and Eastern European subgroup

	ITT		East Asian subgroup		Japanese subgroup		Eastern European subgroup	
Lesions, *n* (%)	Placebo *n* = 113	DMF^a^ *n* = 111	Placebo *n* = 72	DMF^a^ *n* = 70	Placebo *n* = 58	DMF^a^ *n* = 56	Placebo *n* = 41	DMF^a^ *n* = 41
Number of new Gd^+^ lesions from weeks 12–24								
0	44 (39)	81 (73)	28 (39)	55 (79)	24 (41)	45 (80)	16 (39)	26 (63)
1–2	26 (23)	23 (21)	16 (22)	10 (14)	13 (22)	8 (14)	10 (24)	13 (32)
≥ 3	43 (38)	7 (6)	28 (39)	5 (7)	21 (36)	3 (5)	15 (37)	2 (5)
Number of new Gd^+^ lesions from baseline to week 24								
0	35 (31)	58 (52)	21 (29)	38 (54)	17 (29)	30 (54)	14 (34)	20 (49)
1–2	23 (20)	37 (33)	14 (19)	25 (36)	12 (21)	21 (38)	9 (22)	12 (29)
≥ 3	55 (49)	16 (14)	37 (51)	7 (10)	29 (50)	5 (9)	18 (44)	9 (22)
Number of new/newly enlarging T2 hyperintense lesions at week 24								
0	35 (31)	44 (40)	22 (31)	29 (41)	18 (31)	23 (41)	13 (32)	15 (37)
1–2	18 (16)	47 (42)	11 (15)	26 (37)	10 (17)	22 (39)	7 (17)	21 (51)
≥ 3	60 (53)	20 (18)	39 (54)	15 (21)	30 (52)	11 (20)	21 (51)	5 (12)

*Abbreviations: MRI* magnetic resonance imaging, *ITT* intention–to-treat, *Gd*^*+*^ gadolinium-enhancing

^a^DMF, delayed-release DMF

On exploratory clinical endpoints, the relative reduction in ARR with DMF compared with placebo over 24 weeks was 31% in the overall ITT population (0.45 vs 0.65; *p* = 0.1251) and 48% and 47% in the Japanese and East Asian subgroups, respectively. The proportion of patients with relapse over 24 weeks was reduced with DMF treatment compared with placebo by 42% in the overall ITT population (0.21 vs 0.30; *p* = 0.0472) and by 56% and 53% in the Japanese and East Asian subgroups, respectively (Table 2).

### Safety

#### Adverse events

The majority of patients reported ≥1 AE (placebo, 77%; DMF, 86%; Table 4). Most AEs were mild or moderate in severity. Although incidence of nasopharyngitis was higher in Asian subgroups compared to the ITT population and Eastern European subgroup, incidence was similar between patients treated with placebo and DMF in all subgroups (Table 4). AEs that occurred at an incidence ≥2% higher in the DMF group compared to the placebo group included flushing (8% placebo vs 22% DMF), hot flush (<1% vs 6%), diarrhea (5% vs 10%), abdominal pain (0% vs 7%), nausea (5% vs 10%), pruritus (2% vs 7%), and alanine aminotransferase (ALT) increase (2% vs 6%). In the DMF group, the incidence of flushing events (flushing and hot flush) and gastrointestinal (GI) events (diarrhea, nausea, abdominal pain, upper abdominal pain, and vomiting) was highest during the first month of treatment (14% for flushing and 5% for hot flush; 4–9% for individual GI events) and then steadily decreased through the sixth month of treatment (<1% for flushing and 0% for hot flush; 0% for individual GI events). MS relapse was reported more frequently with placebo (31%) compared with DMF (23%).

**Table 4:** Overall summary of AEs (safety population)

	ITT		East Asian subgroup		Japanese subgroup		Eastern European subgroup	
AE, *n* (%)	Placebo *n* = 113	DMF^a^ *n* = 111	Placebo *n* = 72	DMF^a^ *n* = 70	Placebo *n* = 58	DMF^a^ *n* = 56	Placebo *n* = 41	DMF^a^ *n* = 41
Any AE	87 (77)	96 (86)	61 (85)	64 (91)	49 (84)	53 (95)	26 (63)	32 (78)
Mild	50 (44)	58 (52)	31 (43)	38 (54)	22 (38)	29 (52)		
Moderate	35 (31)	32 (29)	28 (39)	24 (34)	26 (45)	22 (39)		
Severe	2 (2)	6 (5)	2 (3)	2 (3)	1 (2)	2 (4)	0	4 (10)
Most frequently reported AEs^b^								
Nasopharyngitis	28 (25)	26 (23)	23 (32)	24 (34)	22 (38)	21 (38)	5 (12)	2 (5)
MS relapse	35 (31)	25 (23)	30 (42)	18 (26)	27 (47)	16 (29)	5 (12)	7 (17)
Flushing	9 (8)	24 (22)	5 (7)	10 (14)	2 (3)	8 (14)	4 (10)	14 (34)
Diarrhea	6 (5)	11 (10)	5 (7)	9 (13)	5 (9)	8 (14)	1 (2)	2 (5)
Nausea	6 (5)	11 (10)	4 (6)	8 (11)	4 (7)	6 (11)	2 (5)	3 (7)
Abdominal pain	0	8 (7)	0	4 (6)	0	4 (7)	0	4 (10)
Pruritus	2 (2)	8 (7)	2 (3)	8 (11)	2 (3)	6 (11)	0	0
ALT increased	2 (2)	7 (6)	2 (3)	6 (9)	2 (3)	6 (11)	1 (2)	2 (5)
Hot flush	1 (< 1)	7 (6)	1 (1)	6 (9)	1 (2)	6 (11)	0	1 (2)
Abdominal pain upper	6 (5)	5 (5)	5 (7)	3 (4)	4 (7)	3 (5)	1 (2)	2 (5)
Upper respiratory tract infection	11 (10)	5 (5)	< 5%	< 5%	< 5%	< 5%	8 (20)	4 (10)
Tonsillitis	2 (2)	1 (< 1)	0	0	0	0	2 (5)	1 (2)
Serious AE	16 (14)	15 (14)	14 (19)	12 (17)	11 (19)	10 (18)	2 (5)	3 (7)
AE leading to discontinuation of study treatment	2 (2)	1 (< 1)	2 (3)	1 (1)	2 (3)	1 (2)	0	0
AE of special interest								
Flushing and related symptoms	10 (9)	33 (30)	6 (8)	17 (24)	3 (5)	14 (25)	4 (10)	16 (39)
GI tolerability AEs	18 (16)	37 (33)	13 (18)	25 (36)	11 (19)	20 (36)	5 (12)	12 (29)
Infections (including potential opportunistic infections)	47 (42)	45 (41)	27 (38)	32 (46)	24 (41)	27 (48)	20 (49)	13 (32)
CV disorders	1 (< 1)	4 (4)	1 (1)	2 (3)	0	2 (4)	0	2 (5)
Potential hepatic disorders	4 (4)	10 (9)	4 (6)	9 (13)	4 (7)	9 (16)	0	1 (2)
Renal disorders	8 (7)	6 (5)	4 (6)	2 (3)	3 (5)	2 (4)	4 (10)	4 (10)
Potential malignancies and malignancies	1 (< 1)	0	1 (1)	0	1 (2)	0	0	0

*Abbreviations: AE* adverse event, *ITT* intention-to-treat, *MS* multiple sclerosis, *ALT* alanine aminotransferase, *GI* gastrointestinal, *CV* cardiovascular

^a^DMF, delayed-release DMF

^b^Incidence ≥5% in ITT placebo and/or DMF group; presented in order of frequency in the ITT DMF group

The overall incidence of serious AEs (SAEs) was 14% for both placebo and DMF. The incidence of individual SAEs was low (<1%) in both groups except for patients with MS relapse (14% placebo, 11% DMF). There were no deaths reported.

A total of 3 patients discontinued treatment due to an AE. Two patients in the placebo group discontinued due to an MS relapse and liver function abnormality, respectively. One patient in the DMF group discontinued due to liver disorders.

Among the AEs of special interest, infections (including potential opportunistic infections), GI tolerability AEs, and flushing and related symptoms were reported most frequently. The incidence of infections was similar between the placebo (42%) and DMF (41%) groups; no potential opportunistic infections or malignancies were reported in DMF-treated patients. The incidence of flushing and related symptoms (9% vs 30%), GI tolerability AEs (16% vs 33%), and potential hepatic disorders (4% vs 9%) was higher in the DMF group compared with the placebo group.

The results of analyses of AEs were broadly consistent across all regional subgroups (Table 4). However, the incidence of flushing and related symptoms was lower in Japanese patients (placebo, 5%; DMF, 25%) and East Asian patients (placebo, 8%; DMF, 24%) compared with Eastern European patients (placebo, 10%; DMF, 39%).

#### Laboratory assessments

In DMF-treated patients, mean white blood cell (WBC) counts decreased by ~3% (from 6.1 × 10^9^/L at baseline to 5.7 × 10^9^/L at week 24) and mean absolute lymphocyte counts (ALCs) decreased by ~ 16% (from 1.7 × 10^9^/L at baseline to 1.4 × 10^9^/L at week 24). Both mean WBC counts and mean ALCs remained within normal limits at all time points. The incidence of abnormal WBC counts or ALC was higher in the DMF group compared with the placebo group (Table 5). No patients with a low WBC count or ALC experienced a serious infection.

**Table 5:** Summary of hematology laboratory abnormalities and maximum post-baseline values for liver enzymes

	ITT		East Asian subgroup		Japanese subgroup		Eastern European subgroup	
Parameter/criterion	Placebo *n* = 113	DMF^a^ *n* = 111	Placebo *n* = 72	DMF^a^ *n* = 70	Placebo *n* = 58	Placebo *n* = 56	Placebo *n* = 41	DMF^a^ *n* = 41
Patients with any post-baseline value for hematology	113	110	72	69	58	55	41	41
WBC, *n* (%)								
<3 × 10^9^/L	2 (2)	14 (13)	1 (1)	9 (13)	1 (2)	8 (15)	1 (2)	5 (12)
Lymphocytes								
<LLN^b^	7 (6)	34 (31)	4 (6)	19 (28)	4 (7)	16 (29)	3 (7)	15 (37)
< 0.8 × 10^9^/L	3 (3)	24 (22)	2 (3)	12 (17)	2 (3)	10 (18)	1 (2)	12 (29)
< 0.5 × 10^9^/L	1 (< 1)	2 (2)	1 (1)	1 (1)	1 (2)	0	0	1 (2)
Patients with any post-baseline value for liver enzymes	113	110	72	69	58	55		
ALT								
> 1 × ULN	16 (14)	35 (32)	12 (17)	21 (30)	10 (17)	20 (36)	4 (10)	14 (34)
≥ 3 × ULN	3 (3)	5 (5)	2 (3)	4 (6)	2 (3)	4 (7)	1 (2)	1 (2)
> 5 × ULN	1 (< 1)	1 (< 1)	1 (1)	1 (1)	1 (2)	1 (2)	0	0
AST								
> 1 × ULN	11 (10)	26 (24)	9 (13)	18 (26)	8 (14)	16 (29)	2 (5)	8 (20)
≥ 3 × ULN	1 (< 1)	1 (< 1)	1 (1)	1 (1)	1 (2)	1 (2)	0	0
> 5 × ULN	0	1 (< 1)	0	1 (1)	0	1 (2)	0	0
ALT/AST ≥3 × ULN concurrent with bilirubin > 2 × ULN	0	0	0	0	0	0	0	0

*Abbreviations: ITT* intention-to-treat, *WBC* white blood cell, *LLN* lower limit of normal, *ALT* alanine aminotransferase, *ULN* upper limit of normal, *AST* aspartate aminotransferase

^a^DMF, delayed-release DMF

^b^0.91 × 10^9^/L

The incidence of elevated liver transaminases (ALT or aspartate aminotransferase [AST]) was higher in the DMF group compared with the placebo group (Table 5). No case met Hy’s law criteria for drug-induced liver injury (ALT/AST levels ≥3 × upper limit of normal [ULN] concurrent with bilirubin level >2 × ULN).

There were no differences between the overall population and the Japanese, East Asian, and Eastern European subgroups in any of the above laboratory assessments (Table 5).

## Discussion

Meta-analyses of randomized trials in RRMS have convincingly demonstrated the correlation between DMT treatment effects on Gd^+^ and T_2_ lesions on MRI and relapses. In addition, effects on MRI lesions over short follow-up periods (6–9 months) can predict the effects on relapses over longer follow-up periods (12–24 months) [15, 16]. We chose radiological measures to serve as primary and secondary endpoints, due to the ability of MRI to detect lesions that might not produce clinical manifestations in the short term [17]. In this RRMS study in which the majority of enrolled patients were from East Asia, DMF 240 mg BID significantly reduced the total number of new Gd^+^ lesions from weeks 12–24 (primary endpoint) compared with placebo. DMF-treated patients also had significantly fewer new/newly enlarging T2 hyperintense lesions at week 24. Analyses of Japanese and East Asian subgroups showed similar results. These data are consistent with those of phase 2 and 3 studies conducted mainly in white patients [1, 2, 14], and thus confirm the efficacy of DMF in patients from East Asia. In addition, these studies clearly demonstrated efficacy of DMF after 12 weeks of treatment [14, 18]. Clinically meaningful reductions in relapse activity were observed in this study and were generally consistent with previous studies [1, 2, 14]. However, clinical endpoints in this study were exploratory, as the study was not powered to detect a statistically significant difference.

Compared with placebo, DMF reduced the ARR by 31% in the overall population. This result was not statistically significant, likely due to the low placebo group relapse rate in the Eastern European patients. The Japanese DMF subgroup also demonstrated a reduced probability of relapse, beginning from week 8. In the Japanese and East Asian subgroups, effect sizes of relapse activity were 48% and 47%, respectively. In addition, the proportion of patients with relapse was significantly reduced in the overall APEX population, and reduced by 56% and 53% in the Japanese and East Asian subgroups, respectively. These results are consistent with findings in the first 24 weeks of DEFINE and CONFIRM, in predominantly white patients with RRMS [18]. In this study, the total number of relapses in Eastern European subjects in the placebo group was unexpectedly low (7 relapses), with a mean adjusted ARR of 0.20. This may be due to the small sample size of this subpopulation and the short treatment duration. However, it is important to note that similar levels of reduction in MRI disease activity were observed in all 3 subpopulations. Additionally, the efficacy of DMF on clinical endpoints including ARR has been clearly demonstrated in the pivotal studies, which included predominantly Caucasian subjects. EDSS changed minimally in both the placebo and the DMF group, as was expected over the 6-month study period (data not shown).

In the study, relapse rate was higher in the placebo group (0.65) vs. DMF group (0.45) (Table 2). Consistently, the proportion of patients receiving treatment with methylprednisolone was higher in the placebo group (24%) vs. DMF group (16%). Therefore, it is highly unlikely the reduction in clinical and radiological disease activity in the DMF group was due to steroid treatment.

The overall safety profile of DMF was consistent with that observed previously [1, 2, 19, 20]. Compared with placebo, DMF was associated with an increased incidence of flushing and related symptoms and GI tolerability AEs, a reduction in mean WBC count and ALC, and an increased incidence of elevated liver transaminases. However, these AEs were mostly mild or modest in severity, and the incidence of flushing and GI events, which was highest during the first month of the study, decreased substantially in subsequent months. In addition, <1% of DMF-treated patients discontinued treatment due to AEs. There was no evidence of an increased risk of infection, cardiovascular disorders, renal disorders, or malignancies related to DMF treatment. Part II of APEX is an ongoing open-label extension trial designed to examine the safety and tolerability of DMF in this population.

The safety profile of DMF was broadly consistent across regional subgroups. No clinically meaningful differences in AEs or laboratory results were identified in the Japanese and East Asian subgroups, including the incidence of lymphopenia and liver function abnormalities. Interestingly, the incidence of flushing and related symptoms was numerically lower in Japanese patients (placebo vs DMF: 5% vs 25%) and East Asian patients (placebo vs DMF: 8% vs 24%) compared with Eastern European patients (placebo vs DMF: 10% vs 39%). In an integrated analysis of DEFINE and CONFIRM, the incidence of flushing also was higher in white patients (placebo [*n* = 687] vs DMF [*n* = 625]: 5% vs 38%) compared with patients of other racial and ethnic backgrounds (placebo [*n* = 116] vs DMF [*n* = 112]: 2% vs 9%). However, the difference between East Asian and white patients observed in the APEX study should be interpreted with caution due to the relatively small sample size.

## Conclusions

In this 6-month study, in which a majority of patients were enrolled from East Asia, the efficacy and safety of DMF were consistent with previous phase 2 and 3 studies with predominantly white patients with MS [1, 2, 14]. The effects of DMF 240 mg BID in the Japanese and East Asian subgroups were similar to those in the overall APEX study population. These results suggest that the strong efficacy and favorable benefit-risk profile of DMF extends to Japanese and other East Asian patients with MS.

## Additional files

Additional file 1: Saida_supplementary material_additional methods. (DOCX 36 kb)

Additional file 2: Saida_supplementary material_IRB table. (DOCX 38 kb)

## Abbreviations

AE: Adverse event
ALC: Absolute lymphocyte count
ALT: Alanine aminotransferase
ARR: Annualized relapse rate
AST: Aspartate aminotransferase
BID: Twice daily
CMQ: Custom Medical Dictionary for Regulatory Activities Query
CV: Cardiovascular
DMF: Delayed-release dimethyl fumarate
EDSS: Expanded Disability Status Scale
Gd^+^: Gadolinium-enhancing
GI: Gastrointestinal
HLGT: High Level Group Term
ITT: Intention-to-treat
MedDRA: Medical Dictionary for Regulatory Activities
MRI: Magnetic resonance imaging
MS: Multiple sclerosis
NMOSD: Neuromyelitis optica spectrum disorder
PT: Preferred Term
RRMS: Relapsing-remitting multiple sclerosis
SAE: Serious adverse event
SMQ: Standardized Medical Dictionary for Regulatory Activities Query
SOC: System Organ Class
ULN: Upper limit of normal
WBC: White blood cell
