# Supplementary material for: A randomized placebo-controlled trial of delayed-release dimethyl fumarate in patients with relapsing-remitting multiple sclerosis from East Asia and other countries
Source: BMC Neurol. 2019 Jan 7;19:5. doi: 10.1186/s12883-018-1220-3 (PMC6322309; doi:10.1186/s12883-018-1220-3)
Supplement: Supplementary file 1 — Saida_supplementary material_additional methods. (DOCX 36 kb) [file 12883_2018_1220_MOESM1_ESM.docx]

# Additional file 1

# Exclusion criteria for contraindicated medication

Patients treated with interferon beta or glatiramer acetate within 3 months before randomization, natalizumab or fingolimod within 6 months before randomization, intravenous steroids within 50 days before randomization, or delayed-release dimethyl fumarate (DMF) or fumaric acid esters at any time before randomization were excluded.

# Randomization and blinding

Patients were registered at the time of consent and randomized at baseline (day 1/enrollment visit), after all screening assessments were completed and after the investigator verified their eligibility. A stratified block randomization procedure was used for Part I, with 5 strata, 1 for each country (block size: 4; 50 blocks per country). Within each country subgroup, patients were randomized to receive DMF or placebo in a 1:1 ratio. Patients who withdrew from the study or discontinued study treatment were not replaced. The date of first treatment was March 29, 2013, and the date of last patient, last visit was June 16, 2015 (end of APEX Part I).

Registration and randomization took place across all investigational sites. At randomization, a centralized interactive voice/web response system assigned a unique 6-digit patient identification number to each patient. The patient’s identification number was used on all of that patient’s case report forms.

DMF and placebo administration was double blind. Placebo capsules matched DMF capsules in size, shape, color, and taste. All patients (including those receiving placebo) were dosed with the same number of capsules twice daily.

# Sensitivity analyses

Three sensitivity analyses were conducted for the primary endpoint (total number of new gadolinium-enhancing [Gd^+^] lesions in weeks 12–24). The first used the Wilcoxon rank-sum test to compare the number of new Gd^+^ lesions between the placebo and DMF groups, which was the method used for this endpoint in the phase 2b study of DMF [[14](#_ENREF_14)]. The mean number of lesions was 4.3 in the placebo group and 1.1 in the DMF group, indicating a reduction of 74% with DMF compared with placebo (*p* < 0.0001).

The second sensitivity analysis excluded patients with outlying lesion values (>30 new Gd^+^ lesions in any post-baseline scan) and patients who tested positive for anti–aquaporin 4 antibody on study (either on day 1 of the study or at the first relapse visit). The mean number of lesions was 4.4 in the placebo group and 0.6 in the DMF group, indicating a reduction of 83% with DMF compared with placebo (*p* < 0.0001).

The third sensitivity analysis assessed the effect of an alternative imputation approach in which the average of valid readings at the visits immediately before and after the visit with the missing value were used to impute the missing value. The mean number of lesions was 3.2 in the placebo group and 0.6 in the DMF group, indicating a reduction of 83% with DMF compared with placebo (*p* < 0.0001).

# Adverse events (AEs) of special interest

AEs of special interest were defined based on Standardized Medical Dictionary for Regulatory Activities (MedDRA) Queries (SMQs), Custom MedDRA Queries (CMQs), System Organ Classes (SOCs), High Level Group Terms (HLGTs), High Level Terms, and/or Preferred Terms (PTs), as appropriate.

## **Flushing and related symptoms**

Flushing and related symptoms were defined by the PTs of flushing, hot flush, erythema, generalized erythema, burning sensation, skin burning sensation, feeling hot, and hyperemia.

## **Gastrointestinal (GI) tolerability AEs**

GI tolerability AEs were defined by PTs in the level 2 subordinate SMQs gastrointestinal nonspecific inflammations, gastrointestinal nonspecific symptoms and therapeutic procedures, and gastrointestinal nonspecific dysfunction.

## **Infections (including potential opportunistic infections)**

Infections (including potential opportunistic infections) were defined by all PTs in the SOC infections and infestations.

## **Cardiovascular (CV) disorders (including ischemic CV disorders)**

CV disorders were defined by PTs in the level 1 SMQ ischemic heart disease, PTs in the level 3 subordinate SMQ ischemic cerebrovascular conditions, cardiac CMQs with primary PTs under the cardiac SOC and secondary PTs of interest not listed under level 1 SMQ ischemic heart disease, and HLGT cardiac and vascular investigations (excluding enzyme tests) excluding duplicate PTs also listed in the ischemic heart disease SMQ.

## **Potential hepatic disorders**

Potential hepatic disorders were defined by PTs in the level 2 subordinate SMQ drug-related hepatic disorder – comprehensive search.

## **Renal disorders**

Renal disorders were defined by subsets of AEs in the renal and urinary disorders and investigations SOCs, and renal function laboratory parameters (eg, blood urea nitrogen, creatinine, beta-2-microglobulin, and microalbumin) deemed medically relevant by the sponsor.

## **Potential malignancies and malignancies**

Potential malignancies were defined by PTs in the level 2 subordinate SMQ malignant or unspecified tumors. AEs identified as potential malignancies based on this SMQ search underwent medical review by the sponsor to identify those events that would be classified as malignancies (ie, malignant neoplasms). In particular, this review sought to exclude any benign neoplasms identified as potential malignancies from being classified as malignancies.
