# Supplementary material for: A randomized placebo-controlled trial of delayed-release dimethyl fumarate in patients with relapsing-remitting multiple sclerosis from East Asia and other countries
Source: BMC Neurol. 2019 Jan 7;19:5. doi: 10.1186/s12883-018-1220-3 (PMC6322309; doi:10.1186/s12883-018-1220-3)
Supplement: Supplementary file 2 — Saida_supplementary material_IRB table. (DOCX 38 kb) [file 12883_2018_1220_MOESM2_ESM.docx]

Listing of Ethics Committees

| Site No. | Name / City of Ethics Committee or Institutional Review Board |
| --- | --- |
| 201 | Koseikai Sone Clinic IRB  Shinjuku‐ku, Tokyo‐To |
| 202 | National Center Hospital, NCNP IRB  Kodaira‐shi, Tokyo‐To |
| 203 | Saitama Medical Center IRB  Kawagoe‐shi, Saitama‐Ken |
| 204 | Chiba University Hospital IRB  Chiba‐shi, Chiba‐Ken |
| 205 | NHO Utano Hospital IRB  Kyoto‐shi, Kyoto‐Fu |
| 206 | NHO Hokkaido Medical Center IRB  Sapporo‐shi, Hokkaido |
| 207 | Tokyotohokeniryokosya Ebara Hospital IRB  Ota‐ku, Tokyo‐To |
| 208 | Sapporo Medical Association IRB  Sapporo‐shi, Hokkaido |
| 209 | National Defense Medical College Hospital IRB  Tokorozawa‐shi, Saitama‐Ken |
| 210 | Iwate Medical University IRB  Morioka‐shi, Iwate‐Ken |
| 211 | Tohoku University Hospital IRB  Sendai‐shi, Miyagi‐Ken |
| 212 | Tsukuba University Hospital IRB  Tsukuba‐shi, Ibaraki‐Ken |
| 213 | Osaka University Hospital IRB  Suita‐shi, Osaka‐Fu |
| 214 | Keio University Hospital IRB  Shinjuku‐ku, Tokyo‐To |
| 215 | Juntendo University Hospital IRB  Bunkyo‐ku, Tokyo‐To |
| 216 | Kyoto University Hospital IRB  Kyoto‐shi, Kyoto‐Fu |
| 217 | Kyushu University Hospital IRB  Fukuoka‐shi, Fukuoka‐Ken |
| 218 | Yamaguchi University Hospital IRB  Ube‐shi, Yamaguchi‐Ken |
| 219 | Niigata University Medical & Dental Hospital IRB  Niigata‐shi, Niigata‐Ken |
| 220 | Ehime University Hospital IRB  Toon‐shi, Ehime‐Ken |
| 221 | Kobe University Hospital IRB  Kobe‐shi, Hyogo‐Ken |
| 222 | Kinki University Hospital IRB  Osakasayama‐shi, Osaka‐Fu |
| 223 | Kanazawa Medical University Hospital IRB  Kahoku‐gun, Ishikawa‐Ken |
| 224 | Tokyo Women's Medical University Hospital IRB  Shinjuku‐ku, Tokyo‐To |
| 226 | Tokyo Women's Medical University Hospital IRB  Shinjuku‐ku, Tokyo‐To |
| 227 | Yokohama City University Hospital IRB  Yokohama‐shi, Kanagawa‐Ken |
| 228 | NHO Asahikawa Medical Center IRB  Asahikawa‐shi, Hokkaido |
| 301 | IRB of National Cancer Center  Goyang‐si, Gyeonggi‐do |
| 302 | IRB of Asan Medical Center  Songpa‐gu, Seoul |
| 303 | IRB of Samsung Medical Center  Seoul |
| 304 | IRB of Seoul National University Hospital  Seoul |
| 305 | Yeungnam University Hospital  Nam‐gu, Daegu |
| 306 | IRB of Korea University Anam Hospital  Seongbuk‐Gu, Seoul |
| 501 | Chang Gung Medical Foundation, Institutional Review Board  Gueishan Township, Taoyuan County |
| 502 | Taipei Veterans General Hospital, Institutional Review Board  Taipei |
| 503 | National Taiwan University Hospital, Research Ethics Committee  Taipei |
| 504 | China Medical University Hospital, Institutional Review Board  Taichung |
| 505 | National Cheng Kung University Hospital, Institutional Review Board  Tainan |
| 701 | Eticka komise u sv. Anny v Brne  Brno |
| 702 | Eticka komise pri Nemocnici Jihlava  Jihlava |
| 703 | Eticka komise Fakultni nemocnice Hradec Kralove  Hradec Kralove |
| 704 | Eticka komise IKEM a FTNsP  Praha |
| 705 | Eticka komise FN a LF UP Olomouc‐LEC  Olomouc |
| 706 | New address:  Privatni ordinace – neurologie; Nestatni zdravotnicke zarizeni  Hradec Kralove  Old address:  Eticka komise  Nachod |
| 707 | Eticka komise Vseobecne fakultni nemocnice v Praze  Praha |
| 708 | Eticka komise nestatniho zdravotnickeho zarizeni MUDR. Sipula  Moravska Ostrava |
| 709 | Eticka komise Fakultni nemocnice Kralovske Vinohrady  Praha |
| 710 | Eticka komise Nemocnice Teplice  Teplice |
| 711 | Eticka komise pro multicentricke klnicke hodnoceni  Praha |
| 802 | Komisja Bioetyczna przy Okregowej Izbie, Lekarskiej w Gdansku  Gdańsk |
| 803 | Komisja Bioetyczna przy Okregowej Izbie, Lekarskiej w Gdansku,  Gdańsk |
| 804 | Komisja Bioetyczna przy Okregowej Izbie, Lekarskiej w Gdansku,  Gdańsk |
| 805 | Komisja Bioetyczna przy Okregowej Izbie, Lekarskiej w Gdansku,  Gdańsk |
| 806 | Komisja Bioetyczna przy Okregowej Izbie, Lekarskiej w Gdansku,  Gdańsk |
| 807 | Komisja Bioetyczna przy Okregowej Izbie, Lekarskiej w Gdansku,  Gdańsk |
| 808 | Komisja Bioetyczna przy Okregowej Izbie, Lekarskiej w Gdansku,  Gdańsk |
| 809 | Komisja Bioetyczna przy Okregowej Izbie, Lekarskiej w Gdansku,  Gdańsk |
| 810 | New address:  Prywatny Gabinet Neurologiczny  Lodz  Old address:  Komisja Bioetyczna przy Okregowej Izbie, Lekarskiej w Gdansku,  Gdańsk |
